# Hybrid asset localization using light fidelity and Bluetooth Low Energy

**DOI:** 10.1371/journal.pone.0274452

**Published:** 2022-09-29

**Authors:** Lamya Albraheem, Haifa Alshathri, Raghad Alsheddi, Ruba Alotaibi, Ghaida Alkharashi

**Affiliations:** Department of Information Technology, College of Computer and Information Science, King Saud University, Riyadh, Saudi Arabia; Universiti Malaya, MALAYSIA

## Abstract

Recently, there has been increasing interest in the field of indoor localization. This field of research can facilitate building and asset management. Although there are different technologies that can be used for localization, there are many limitations that need to be improved, and therefore there is a need to explore new technologies and alternatives that can improve indoor localization. It has been proven that visible light can be used to transfer data. A German physicist, Harald Haas, introduced the term “Li-Fi”, which stands for “light fidelity”, as a new technology that uses light as a medium to deliver data. Accordingly, in this study, we have proposed a hybrid asset localization system using Li-Fi and Bluetooth Low Energy (BLE). This system utilizes light-emitting diodes (LEDs) and BLE tags to detect the locations of assets in a smart building with the support of crowdsourcing technology. The system can make the management, maintenance, and localization process of equipment inside the buildings more easier. To achieve the required, the paper provides a comparison between different applications that have been developed for indoor localization using Li-Fi technology in order to highlight the limitations that need more improvement. The proposed system consists of a web-based administrator panel that allows the administrator to manage maps, assets, tags, LED lamps, and maintenance requests, as well as a mobile application that enables the user to locate, search and view asset information. In addition, the mobile application performs the process of crowdsourcing to update the assets’ locations. We experimentally explore the system’s functionalities and the results show that the system can accurately localize assets, and can detect Li-Fi signals from 55 lx and above within a range of 1.5 m. In addition, the BLE stickers can be detected up to 7 meters away, however, the crowdsourcing process to update the asset location is performed if the distance between the mobile application and the asset is less than or equal 1 m which gives accurate results.

## 1. Introduction

The research community has shown increased interest in the field of indoor localization and there are many challenges that should be considered, e.g., the presence of noise and signal interference [1]. One of the most well-known technologies used for localization is global positioning system (GPS) which provides highly accurate locations in an outdoor environment; however, it is not a good choice for indoor areas because of the GPS signal attenuation through building walls and multipath propagation, and therefore there is a need to find a new technology that attempts to address these issues. There are many technologies such as radio frequency identification (RFID), near field communication (NFC) and ZigBee that can be used for localization; however, these technologies have different limitations and issues, and they can be affected by the indoor environment and noise [2]. Although RFID has many features, its use is not widespread on the commodity of user devices because of the cost of a RFID reader. Additionally, most new smartphones have NFC technology, but they work only in very close proximity, making them an inappropriate choice for some applications. ZigBee supports low-power communication; however, it is not widespread in the smartphone and mobile device industry.

One of the latest indoor localization technologies is light fidelity (Li-Fi). It was discovered, in 2011, by a German physicist Harald Hass and refers to visible light communication (VLC) technology that uses light as a medium to deliver high-speed communication in which light-emitting diodes (LEDs) are used as a carrier signal. It also has a much broader spectrum for transmission as compared with conventional methods of wireless communications that rely on radio waves, such as in Wi-Fi. In addition, it transmits the data much faster and has more flexibility than any other technologies [3]. Since there is enormous interest from the research community with respect to using Li-Fi for indoor localization, it is important to work with this technology to support this research field [4].

In addition, it should also be mentioned that Bluetooth Low Energy (BLE) has been used recently to support an indoor localization system. BLE is a low-power wireless technology which was designed for short-range communication to reduce the cost of energy and delay and to increase the performance of discovery. In addition, it has many features such as small size, security, ultra-low power which enables a coin cell to operate over a long period of time [5], and it covers 70–100 meters efficiency with 24 Mbps data rate [1]. BLE has different states such as standby, scanning, advertising, initiating, and connection; however, in indoor localization, the most attractive states are the advertising and scanning states. On the one hand, in the advertising state, a device can detect other devices that are in the same range, in which advertising packages depend on the search type. On the other hand, in the scanning state, a device starts listening to advertising packets [5].

Recent studies have developed different approaches that used hybrid technologies which can improve indoor localization; however, there are no studies that have focused on using Li-Fi and BLE in order to localize the assets in buildings. Therefore, the aim of this study was to contribute by filling the gap that exists in previous studies. We propose a hybrid asset localization approach using Li-Fi and BLE with support of crowdsourcing technology. Crowdsourcing is defined as a new method for collecting information from a crowd or a large number of people based on a collaborative activity. The concept of crowdsourcing involves sharing tasks and helping people to obtain more experience by involving the crowd, mostly online. It can be used as an effective solution for gathering location-related network data that can support location-based services. In addition, due to the increase in smartphone users, each user can act as a contributor in crowdsourcing by utilizing the built-in sensors of smartphones. For example, in a smart building, crowdsourcing is applied by using the smartphones of visitors and employees in the building to collect information [6], and accordingly, use crowdsourcing to support a proposed solution or common goal. The proposed real-time location system can manage assets efficiently. As an example, in healthcare field there is a need to track and localize medical equipment, patients, staff and assets which can reduce costs, enhance the work process and improving the quality of services.

The contributions from this study are the following:

- A review and comparison of different applications that have been developed for indoor localization using Li-Fi technology, highlighting the limitations that need more improvement.
- Developing a Li-Fi-based system for indoor localization that use combination of three technologies, i.e., Li-Fi, BLE, and crowdsourcing.
- An experimental study of a real-world case study to test the functionality of the proposed system and report the results. The admin can use the web panel to manage the assets, LEDs, BLE tags, and maintenance request. While the user or maintenance technician can locate assets, and receive maintenance requests.

The remainder of this paper is organized as follows: In Section 2, we briefly present related studies found in the literature; in Section 3, we review related studies on similar applications and compare them with the presented application; the proposed solution with the hardware and software specification is presented in Section 4; in Sections 5 and 6, we describe and discuss an experiment based on a real-world case study and report the results; finally, we state our conclusions and present future work in Section 7.

## 2. Light fidelity technology

In 2011, Harald Haas introduced Li-Fi technology, which is a VLC technology that uses light-emitting diodes (LEDs) for data transmission and illumination. It is 10,000 times faster than Wi-Fi, with a speed of up to 250 gigabits per second [7]. In addition, Wi-Fi uses radio frequency for communication which can affect the signals and can cause interference and high latency problems with other radio frequency signals such as in aircraft, hospitals and military facilities where there is a sensitivity from the electromagnetic radiation. Li-Fi could be a better solution for overcoming the above-mentioned limitations because of its high bandwidth and its resistance to interference from electromagnetic radiation [8]. VLC systems utilize visible light that occupies the spectrum from 380 to 750 nm corresponding to a frequency spectrum of 430 to 790 THz [9]. By changing the flicker rate of the LED, the frequency of light can be modified by encoding different data channels using blue, red and green LEDs which gives the maximum speed of 10 Gbps. A VLC receiver receives signals only if they are in the same room with the transmitter, therefore, receivers outside the room of the VLC source are not able to receive the signals [10].

Li-Fi has many advantages and benefits, including [11]:

1. LEDs consume less energy and have a low cost.
2. Information access is possible at a very high speed and security.
3. Universal use of this technology means that every light bulb is an open data source.
4. A visible light spectrum is 10,000 times wider than the radio waves.
5. Li-Fi has wider bandwidth than radio waves.
6. LEDs can be turned ON and OFF quickly.

The architecture of a Li-Fi system is made of two important components, i.e., LED bulbs and photodetectors (PDs). PDs and some other components which are used for data reception are integrated into what is called a Li-Fi dongle. The light intensity through a Li-Fi system varies at a very high rate, and therefore it is not visible to the human eye. Similar to Wi-Fi, Li-Fi transmits and receives data electromagnetically [12].

An LED is a semiconductor device that emits light, and it is the core of Li-Fi technology. In Li-Fi, the role of a basic LED bulb is to act as a data transmitter if it is equipped with a microchip that can simultaneously illuminate and transmit data. When it encodes the data into light, it changes the intensity of the LED to transmit data. Therefore, when the LED is switched ON it is transmitting as “1”, when the LED is switched OFF it is transmitting as “0”, and when there is a combination of 1s and 0s it is actually transmitting various strings of data. All of this is achieved in a highspeed flickering that cannot be seen by the human eye [12]. The Li-Fi dongle is considered to be a receiver and is actually receiving the transmitted data. We have a PD that detects the light, and then converts the light into an electrical signal. The PD registers binary values of 1 if the LED is ON, and 0 if the LED is OFF. The data are continuously received and processed and sent to the user [12].

## 3. Related works

There are three indoor localization techniques, i.e., trilateration, fingerprint, and proximity. First, the trilateration technique locates an asset based on its distance from at least three reference points. Second, the fingerprint technique consists of two modes—training (offline) and tracking (online) modes. In the training mode, a rich site summary (RSS) is used to obtain information about the area and the location of access points (AP) and saves this information in a database. In the tracking mode, the real-time RSS is matched with the one stored in the database to detect the location. Third, the proximity technique determines the location based on its range according to a known station or a close AP [13]. In this section, different studies and applications that have been conducted on indoor localization fields are discussed. In addition, a comparison between different applications is presented to identify the best features that should be considered in the design of an efficient system that uses Li-Fi technology.

### 3.1 Research studies

Huang et al. (2016) conducted a study on "refining Wi-Fi based indoor localization with Li-Fi assisted model calibration in smart buildings". This study aimed to develop a system to track and locate users inside a building using their smartphone. Whenever a user entered the building, his smartphone received Wi-Fi signals and the software “LR-CODE READER” received the Li-Fi signals from a Li-Fi lamp. As a result of detecting the Li-Fi signals successfully, the identifier of the lamp was known. Then, the user location was detected based on the distance between the Wi-Fi access points and the identified Li-Fi lamp. It continuously computed the distance if the user was moving. In addition, the distance was computed based on received signal strength indication (RSSI) of Wi-Fi signals from three access points and a calibrated coefficient value that was based on the Li-Fi distance. Therefore, the proposed solution in this study can be classified as a type of trilateration technique. As a result, this study improved the accuracy of detecting the indoor locations by using a hybrid technology that consisted of Wi-Fi and Li-Fi [14].

In 2016, Chen et al. proposed "a crowdsourcing indoor localization algorithm via optical camera on a smartphone assisted by Wi-Fi fingerprint". This system used Wi-Fi fingerprint based on a k-weighted nearest neighbors (KWNN) algorithm, the camera and the orientation sensor on a smartphone. In the offline mode, the smartphone scans the area to identify APs at different reference points (RP) with its RSSI values and stores them in the database; moreover, it stores images with the corresponding orientation data. In the online mode, the user takes a picture with the corresponding orientation and the system performs the image matching based on the k-nearest neighbors. Furthermore, the user can upload the results to a database to improve the matching process. This system uses crowdsourcing to update the database and this algorithm improves the accuracy and stability of positioning; however, this system has some disadvantages such as image quality which can be affected by the light. If the image has not been effectively recognized, the system does not perform well [15].

Motamedi et al. (2013) conducted a study on "localization of RFID-equipped assets during the operation phase of facilities". This study used RFID tags that attached to assets, and another type of tags that attached to specific locations such as the entrance of each room. These tags are passive RFID, therefore, there is a need to use an RFID reader to scan the area and get the information about all tags that attached to near assets, and their distance to the location tags. to find an asset, the request is sent through a software application that checks if the targeted asset is in the collected information sent from the RFID reader. If the asset is found, the location appears in the floor plan, otherwise the application recommends moving to another location [16].

Kao et al. (2017) conducted a study on "hybrid indoor positioning for asset tracking using Bluetooth low energy and Wi-Fi ". The proposed system gives the user the ability to track assets and update its location continuously. It uses Wi-Fi fingerprinting to locate mobile devices and the BLE trilateration technique(18) to accurately locate the asset position. It works as follows: In the Wi-Fi offline mode, the Wi-Fi radio map is built after the site survey is performed. In the online mode, the location of the user’s mobile device is estimated by comparing the received fingerprint with the stored one in the database using a k-nearest neighbor algorithm to determine the user probable position (PP). Then, to localize the asset, the detected BLE universally unique identifier (UUID) and RSSI of the BLE beacon are stored in the database, and by using the trilateration technique which depends on three PP users that gained in the same period of time, the location is accurately positioned [17].

To deal with the problem of nonlinearity of RSSI measurements, Jondhale et al. proposed the Generalized Regression Neural Network (GRNN) model to give an estimation for moving target. The model is trained by RSSI measurements values that received from different locations of BLE nodes. Then the model is tested to estimate the location of moving target. The location is improved later using Kalman Filter (KF) framework [18]. In 2021, in order to improve the localization accuracy of trilateration technique. Jondhale et al. proposed a model called Trilateration Centroid Generalized Regression Neural Network. It used the neural network to estimate the location by considering the RSS measurements and the centroid values. The results show that the performance of the proposed technique is better than trilateration, and GRNN algorithm [19].

In 2022, an RSS-based target localization technique is proposed. It aims to improve the RSS-based systems and cover the limitations of existing techniques such as trilateration. The proposed model is based on support vector regression (SVR) and Kalman filter (KF). The model require training phase to train the model of different RSS inputs with their locations output. On the time of online estimation, the model needs three RSS measurements to estimate the locations of a mobile target. After testing the model using different experiments, it is shown that the proposed algorithm provide better performance in comparing of other indoor localization techniques such as trilateration- and GRNN-based localization [20].

Furthermore, Yang et al. proposed a new localization approach which is called ultra-wideband (UWB) based unmanned aerial vehicle (UAV) localization approach. The main aim of this approach is localizing the UAV in some environments that have difficulty to be accessed by human. The proposed approach is more accurate than the traditional methods of localization such as GPS or vision based techniques. It consist of two methods which are the two-way time-of-flight (TW-TOF) localization scheme and the maximum likelihood estimation (MLE) method. After conducting different experiments, its shown that the average localization error is under 0.2 m [21].

In 2020, a new indoor target intrusion sensing technique is proposed. The main issue of the existing techniques is in the manual constructing of radio map that used for indoor intrusion detection and localization. Therefore, this paper proposed a new ray-aided generative adversarial model (RaGAM) that can overcome this problem and build the radio map automatically. This model can find the difference of the received signal strength (RSS) in normal and intrusion environments to be used for building the radio map. The radio map is used to prepare training set of probabilistic neural network (PNN). This model will be used later to identify if the newly RSS data are intrusion or not. After testing the proposed model, it is shown that the results are accurate and the computation cost is reduced [22]. For more information about indoor localization, this book covers the fundamental of wireless sensors networks. It reviews also the recent studies, architecture and techniques in localization and tracking using wireless sensor network. It presents different techniques such as Trilateration-Based techniques, KF-based techniques, and Supervised learning architecture. It shows also some of the implementation codes to use these techniques [23].

The above-mentioned related works used different localization techniques which can be categorized into trilateration, fingerprint and proximity techniques. Additionally, these studies have usually used hybrid systems, gaining the advantages of more than one technology for more accurate results. The technologies used included Wi-Fi and Li-Fi, crowdsourcing and Wi-Fi, RFID, as well as BLE and Wi-Fi. As mentioned before, there are many technologies that can be used for localization. However, these technologies have different limitations and issues, they may be affected by the Indoor environment and noise. These limitations can be reduced using Li-Fi technology which provides a combination of low interference and high bandwidth. Since there is a huge interest in the research community with using Li-Fi for indoor localization, it is worth to work with this technology to support this research field [24]. Furthermore, the using of Lifi-based proximity method can improve the accuracy of indoor localization since the signals of light cannot passed through walls, so the light bulbs can be used as identifiers of specific locations. Consequently, it is recommended to conduct a study on the ability of hybrid technologies, such as Li-Fi and BLE, to locate assets in smart buildings. In addition, crowdsourcing can be used as supported technology to update the asset locations in the system. This can be contribution to the research community and fill the gap in the previous studies. In addition, the crowdsourcing can be used as supported technology to update the assets locations in the system.

### 3.2. Applications

Lunera is a company with a mobile application for locating assets using Wi-Fi and BLE. The company’s Lunera Smart Lamp is an integrated module that consists of four components a Wi-Fi radio, a Bluetooth radio, an ambient light sensor, and a microprocessor. The Wi-Fi radio provides a secure connection to the cloud. The Bluetooth radio can be used to connect any Bluetooth *device* in range to form a local network. This smart lamp can be used as a broadcasting beacon to other devices that are listening. In addition, it can be a listener for the devices that want to send their locations. The application provides the location of the users on a building map in real time using triangulation technology and also allows the user to perform different functions such as indoor wayfinding and searching for a specific asset. The manager should attach Beacons to important assets and can also manage a map of a facility, place fixtures, lamps, sensors, define zones and monitor the location of the asset on the map [25, 26].

Aswaaq reach is an android application belonging to Aswaaq in Dubai, United Arab Emirates. It was developed by Philips, Aisle411, AlphaData, ValueLabs and Aswaaq to make the shopping experience easier [27, 28]. The application provides the ability to scan an item’s barcode and adds it to a shopping list from home. In the store, the shopper receives optimal route guidance to all items on their shopping list, and it can also inform the user of nearby discounts and even get recipe suggestions based on their shopping list. When the shopper enters the store, the lamp sends a unique code to the front-facing camera of a shopper’s smartphone, and then the system identifies this code and determines the exact position of the phone on the store’s map. In addition, the application assists a manager by capturing real-time data, allowing analytics of shopper traffic and behavior, and optimizing operations [28].

E. Leclerc is a hypermarket in France that uses Li-Fi LEDs from the OLEDCOMM company to obtain accurate data about their daily shopping activity at the market. Instead of using the camera in a consumer’s smartphone to receive the signal from LEDs, they use a Li-Fi receiver built into the cart to track the shopper’s cart in real time and send data to the cloud through smartphones using BLE technology. The shoppers receive notifications and coupons about the products depending on their locations; moreover, they can search for specific products. This application provides the manager with the ability to view a heatmap of the market and to identify crowded areas at different times and days; also, it can visualize and analyze the path of shopping carts [29].

Régie autonome des transports parisiens (RATP) is a public transport operator in Paris that offers an indoor geolocation service. The user uses a dongle on his smartphone to receive the data signal from LED sources and each LED light tube is embedded with a GeoLifi modem. Through the RATP application, the user can identify a precise indoor geolocation in the La Défense metro station; it also helps impaired persons to indicate their location from some LED lamps by receiving content and converting it to speech [30].

The comparison of the applications presented in Table 1 demonstrates that, on the one hand, most applications aim to find the location of the user, since they can detect users’ locations using their phones which needs less hardware. On the other hand, it is more of a challenge to find the asset (i.e., equipment) than the user, since asset localization requires extra hardware such as identification tags. Asset tracking is one application that targets asset localization, with Wi-Fi and BLE technologies to localize the asset using BLE Smart lamps, BLE tags and Smartphones, where the location is computed based on the triangulation technique to detect the asset location.

**Table 1:** Comparisons among similar applications.

Features		Applications				
		The proposed application	Asset Tracking	Aswaaq reach	E. Leclerc	RATP
General	Platforms	Android	Android/iOS	Android	Android/iOS	-
	Company	Proposed system	Lunera	Philips, Aisle411, Alphadata, Valuelabs And Aswaaq	Oledcomm and E. Leclerc	Oledcomm and RATP
	Languages	English	English	Arabic/English	French	French
	Technologies	Li-Fi, BLE	Wi-Fi, BLE	Li-Fi, Barcode	Li-Fi	Li-Fi
	Hardware	LEDs, BLE tags, Smartphone	BLE Smart lamps, BLE tags, Smartphone	LEDs, Smartphone	Li-Fi receiver, LEDs	Dongle, LEDs, Smartphone
	Purposes	Asset localization	User and asset localization	User localization	Trolley tracking	User localization
Admin	Manage the LED	Yes	Yes	Yes	Yes	Yes
	Manage the tag	Yes	Yes	No	Yes	No
	Manage the asset	Yes	Yes	Yes	No	No
	Manage building Map	Yes	Yes	Yes	Yes	Yes
	View user feedback	Yes	No	No	No	No
User	Search an asset	Yes	Yes	Yes	No	No
	View Map and location of asset	Yes	Yes	Yes	No	No
	Manage/view comments	Yes	No	No	No	No
	View asset information	Yes	No	Yes	Yes	No

Accordingly, based on the comparisons, we have developed an asset indoor localization system using Li-Fi and BLE which we have called “illumication”. It aims at providing users with asset localization in smart buildings and helping them to find their assets utilizing the technology of Li-Fi. It provides a variety of functions to the administrator and the end user. The administrator can add and manage maps and places on that map; furthermore, new assets can be added and managed in addition to the LEDs and BLE tags, as well the user’s feedback about the assets can be viewed. In addition, the proposed system enables the user to view the map according to the range of the LED, to view the assets on the map and to search for specific assets. In addition, the system can be updated using the users’ mobile phones which represent the crowdsourcing component.

## 4. Proposed solution

### 4.1 System architecture

**Fig 1:**
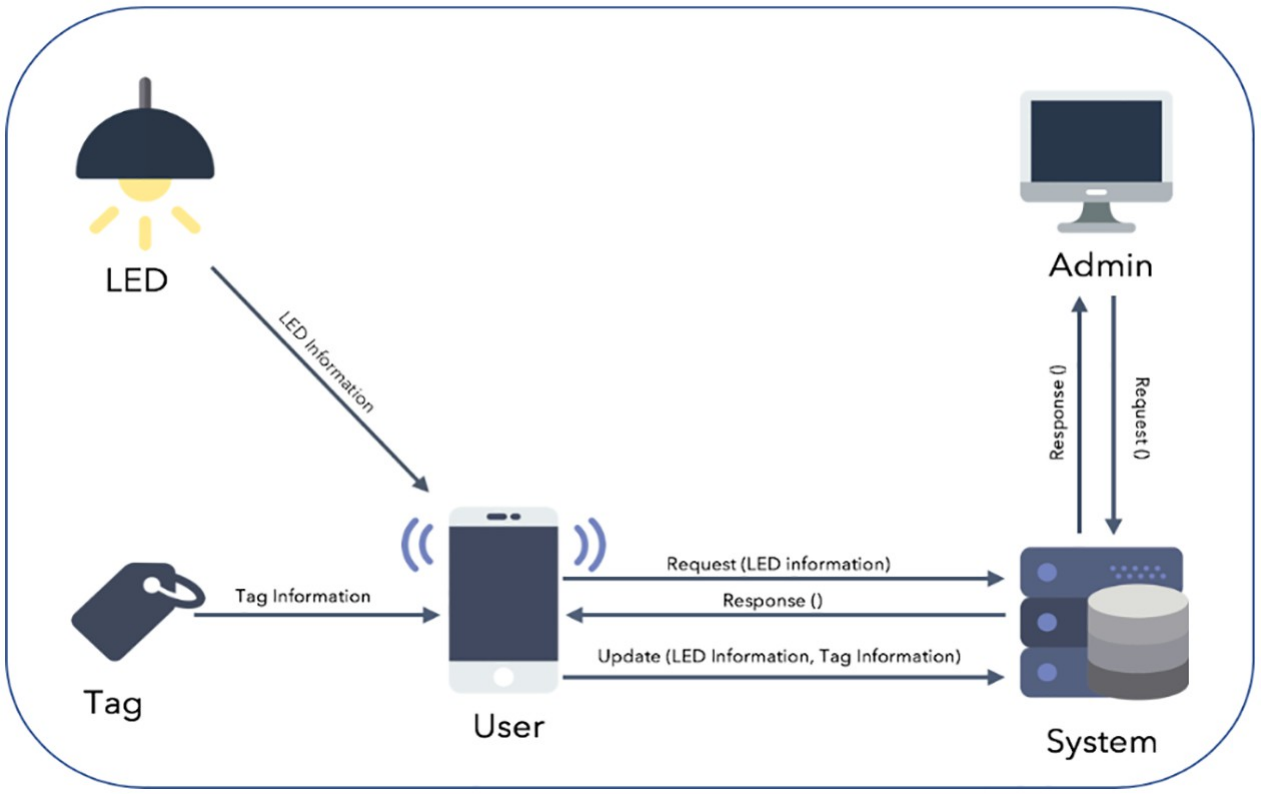
The proposed solution.

The proposed solution, in this study, is a hybrid asset localization approach using Li-Fi and BLE with support of crowdsourcing. The main processes of the system are performing the data acquisition, processing and the analysis of the indoor position data. The components of the system, as shown in Fig 1, are LED lights in the building, BLE tags that are attached to assets and used for identification, and a mobile application developed to find the location of assets in a building and a web-based panel that can manage the systems’ information. The system works as follows: The administrator manages LED lamps, assets and BLE tags present in the building using a web panel. In order to utilize the LEDs to send the location information using light medium to the mobile application, there is a need to modify the light source and to use the modulation technique to send the location codeword to the system. The mobile application can detect the locations of the assets using the information sent by the LED lamp to update the asset locations in the server.

**Fig 2:**
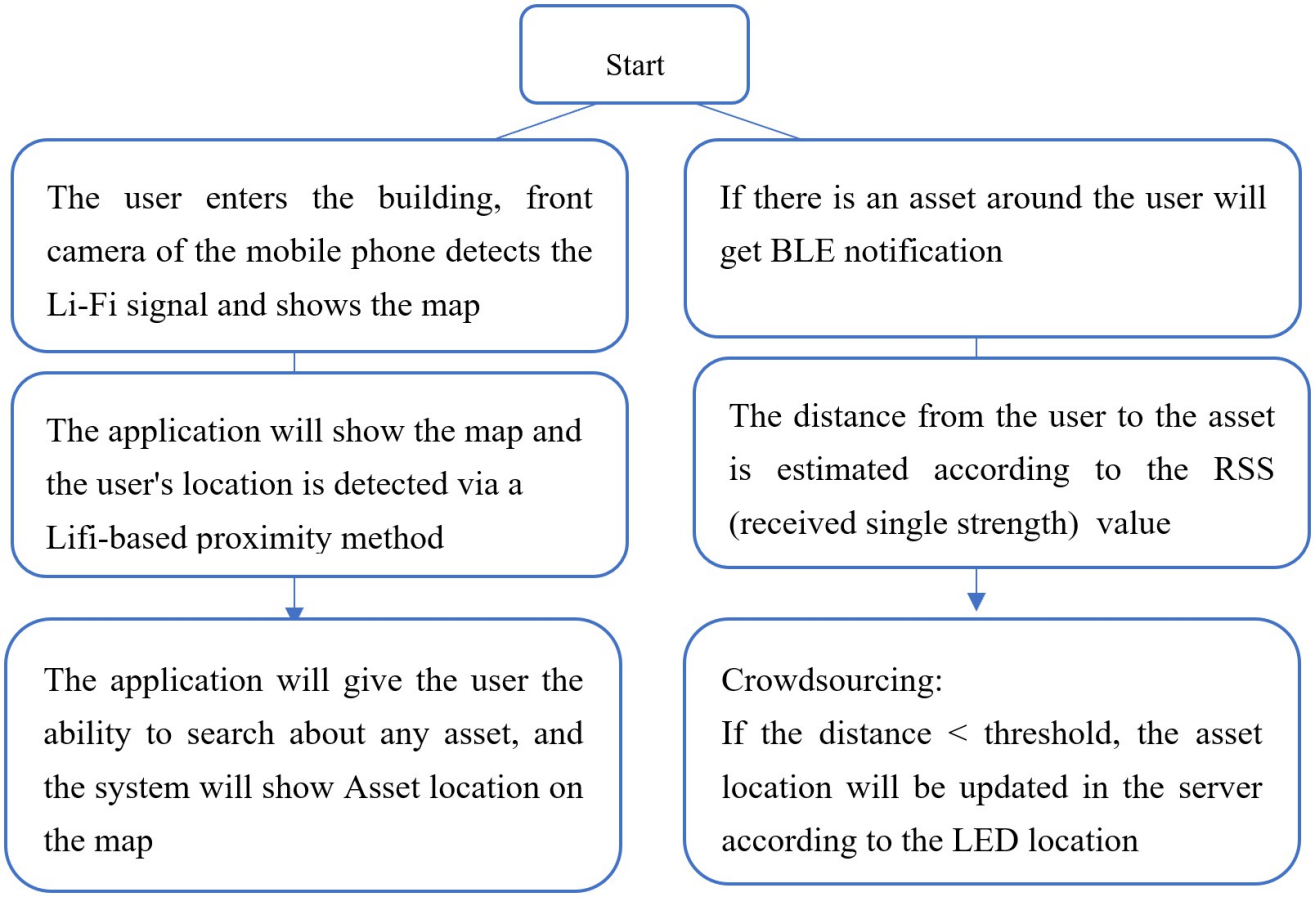
Hybrid asset localization approach of Lifi and BLE.

Then, to update the locations of assets in the server, the LED lamp and the asset tag send their information to the mobile applications of people who walk around inside the building and the locations of the assets are updated according to the LED location if the received signal strength of an asset is less than 1 meter. The updating process is done automatically using mobile phones of users—called crowdsource localization. The steps of the proposed solution are presented in Fig 2.

As shown in Fig 2, when the user enters the building, the front camera of his mobile phone will detect the light signals that received from the nearest bulbs, which could be above the user. The mobile application can make required demodulation to get the codeword that used as an identifier to the current location. The map of the building with the user’s location will be presented to the user using the mobile application. When the user want to search about any asset, the system can show the asset location on the map. This location is estimated periodically and updated using crowdsourcing technique. The process of estimating and updating assets will be done automatically in the background of the application. If the user receives a BLE signal of any asset, so the identifier of the asset and RSSI measurements will be available. The distance from the user to asset will be estimated based on RSSI value, if the distance is less than threshold value, the asset location will be updated according to the location of the nearest LED bulbs.

### 4.2 System analysis

**Fig 3:**
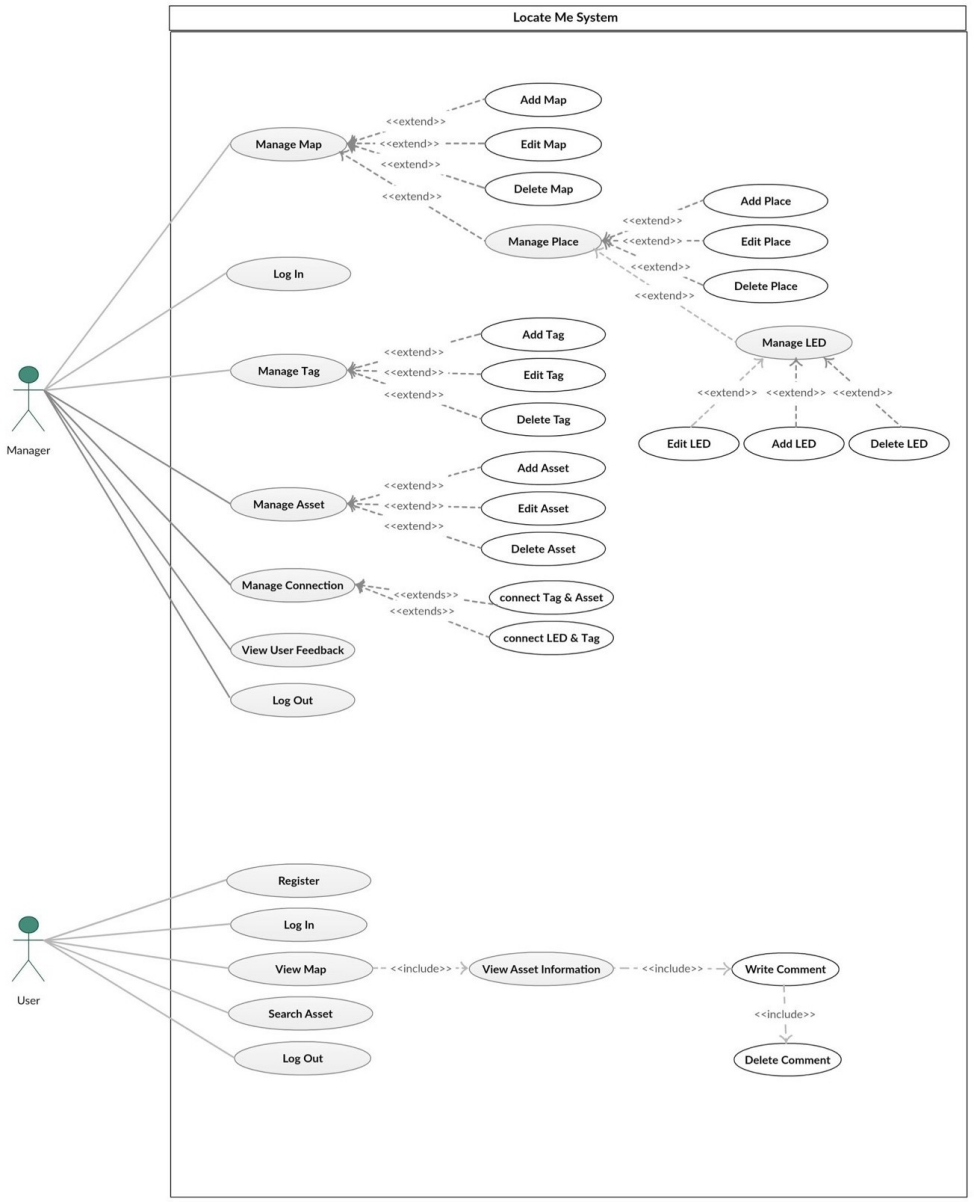
Use case diagram.

The main goal of this project is developing an Assets Localization System using Light Fidelity technology. As shown in Fig 3, this system will utilize the LED lights to detect the locations of available assets in a smart building. It allows the admin to manage maps, assets BLE tags and LED lamps and also make the user able to search, identify and share the location information of assets.

## 5. System implementation

**Fig 4:**
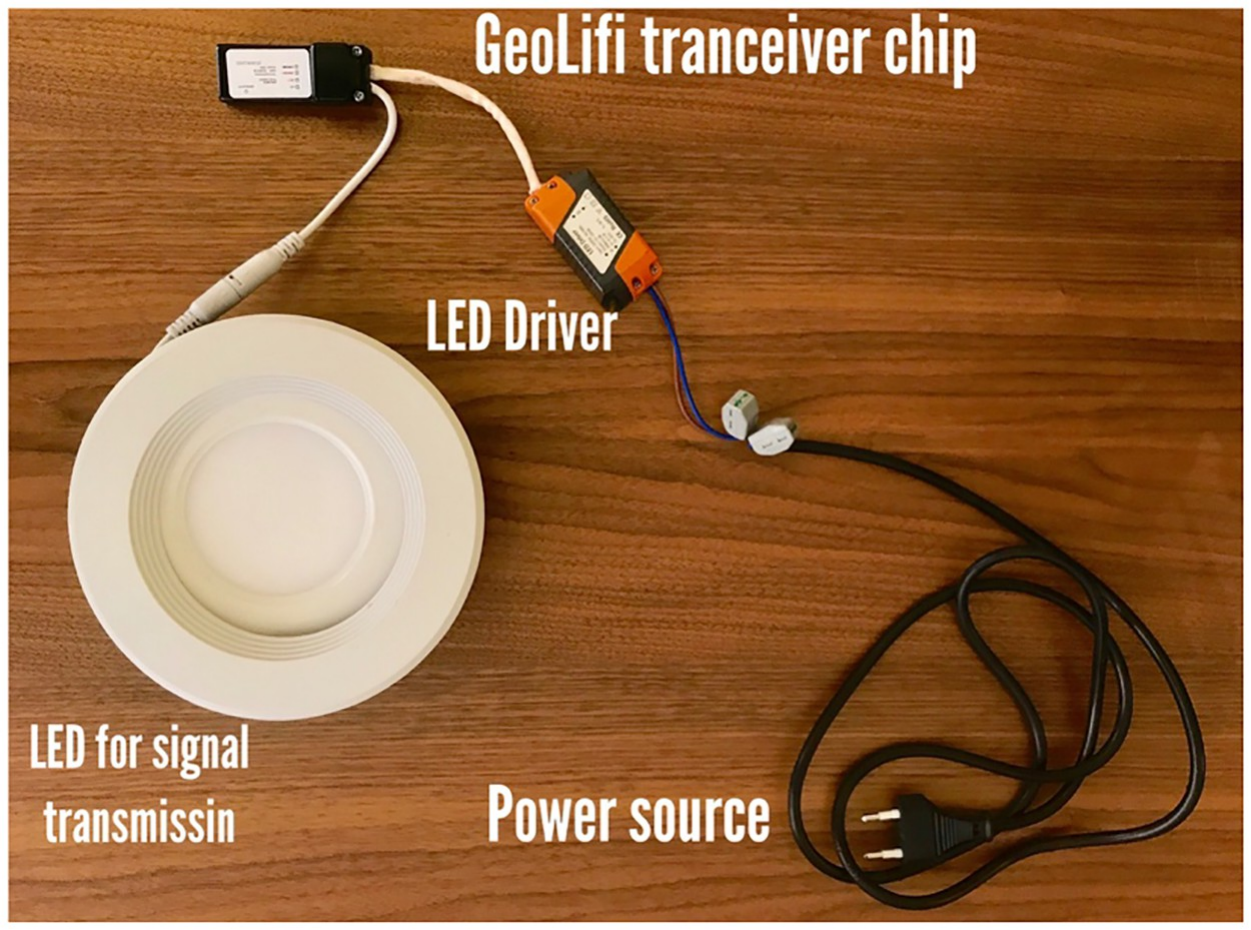
GeoLiFi development kit (camera modulation).

In this section, we describe the system’s hardware which includes LED lights and BLE tags. For Li-Fi, there are different modulation approaches for the design of the LED such as the modulation approach in which receiving the signal is completely dependent on a smartphone’s camera. The hardware used to transmit the Li-Fi signal is shown in Fig 4, which consists of the following four basic components: a power source, an LED driver, a GeoLiFi transceiver chip and the LED. The first component, the power source, is the energy source for the LED. The second component, the LED driver, is responsible for converting a stream of bits to signals for turning the LED on and off. The third component, the GeoLiFi transceiver chip, which is the key component of the LI-Fi LED technology, receives the data from the LED driver, and then sends the data to the LED. The fourth component, the LED, is a semiconductor light source for signal transmitting.

It is recommended to use LED lighting embedded with a GEOLIFI^®^ Module [31] for indoor positioning. These modems can be easily installed and maintained, and they can be connected to an external lamp or integrated to existing lamps.

For the Bluetooth tags, Estimote Stickers (Nearables) [32] are one of the famous products that can act as Bluetooth Smart beacons. They are characterized by being small in size, and therefore are a good choice for identifying assets. They consist of a battery-powered ARM CPU equipped with an accelerometer, temperature sensor, and a Bluetooth Smart radio. The default podcasting power is –12 dbm which results in a range of up to seven meters which helps to locate the asset accurately. The advertising interval, which is controlled by an adaptive algorithm, is 2.6 seconds, if the tags are static and the value is lower when the tags are in motion.

The web panel is developed using Brackets Adobe 1.12, phpMyAdmin, and MySQL for the database. For the mobile application, the Lifi SDK [25] is used to interact with LED lamps, BLE tags SDK [26] and also Xcode to develop an iOS mobile application.

## 6. The experiment

The illumication system is designed to support location-based systems, by providing indoor localization service in smart buildings using Li-Fi technology and the building lights. illumication is deployed in many smart buildings and, for this experiment, the College of Computer and Information Science (CCIS) at King Saud University (KSU) was chosen as the building for the Li-Fi LED installation.

The following three phases must be accomplished for locating the assets using Li-Fi and BLE technologies:

1. Installation and Deployment Phase: The actual installation of the Li-Fi LEDs in the building and placing the Bluetooth tags into the assets.
2. Execution Phase: The mapping between the actual location of LEDs to the logical location in the system and connecting the tag related to the assets to the LED.
3. Testing Phase: The asset localization process is tested by using the mobile application in different scenarios.

### 6.1. Installation and deployment phase

The CCIS building consists of four main floors and each floor has different assets such as chairs, printers and computers; therefore, four different maps are required. In each map, multiple Li-Fi LEDs have been installed and different assets have been connected to tags; in total, CCIS has been occupied with 10 tags and 10 LEDs, for this experiment. The Li-Fi LEDs used in this experiment are manufactured by Oledcomm, namely GEOLIFI^®^ Module for indoor positioning. The installation and deployment involved integrating a transceiver chip in most LEDs in the CCIS building; the transceiver chip can be easily used with most kinds of LED lightings if they meet the chip specifications. Outputs are directly connected to the LED and input connectors are connected to the LED driver, which is itself connected to the power source to supply all the system. Each asset needs to be identified and detected by a BLE tag. Therefore, BLE stickers from Estimote are placed on all assets which turn the items into smart objects. The specifications of the hardware that used for this experiment are presented below in Table 2.

**Table 2:** Experiment parameters.

Required hardware	Specifications
**LED lamps, oldecomm** [31]	
Power (W)	14,8 W
Voltage (V)	234,7
Receiver	Phone’s camera
**BLE stickers, Estimote** [32]	
broadcasting power	–12 dbm
range of up to	7 meters
advertising interval	2.6 seconds

### 6.2. Execution phase

**Fig 5:**
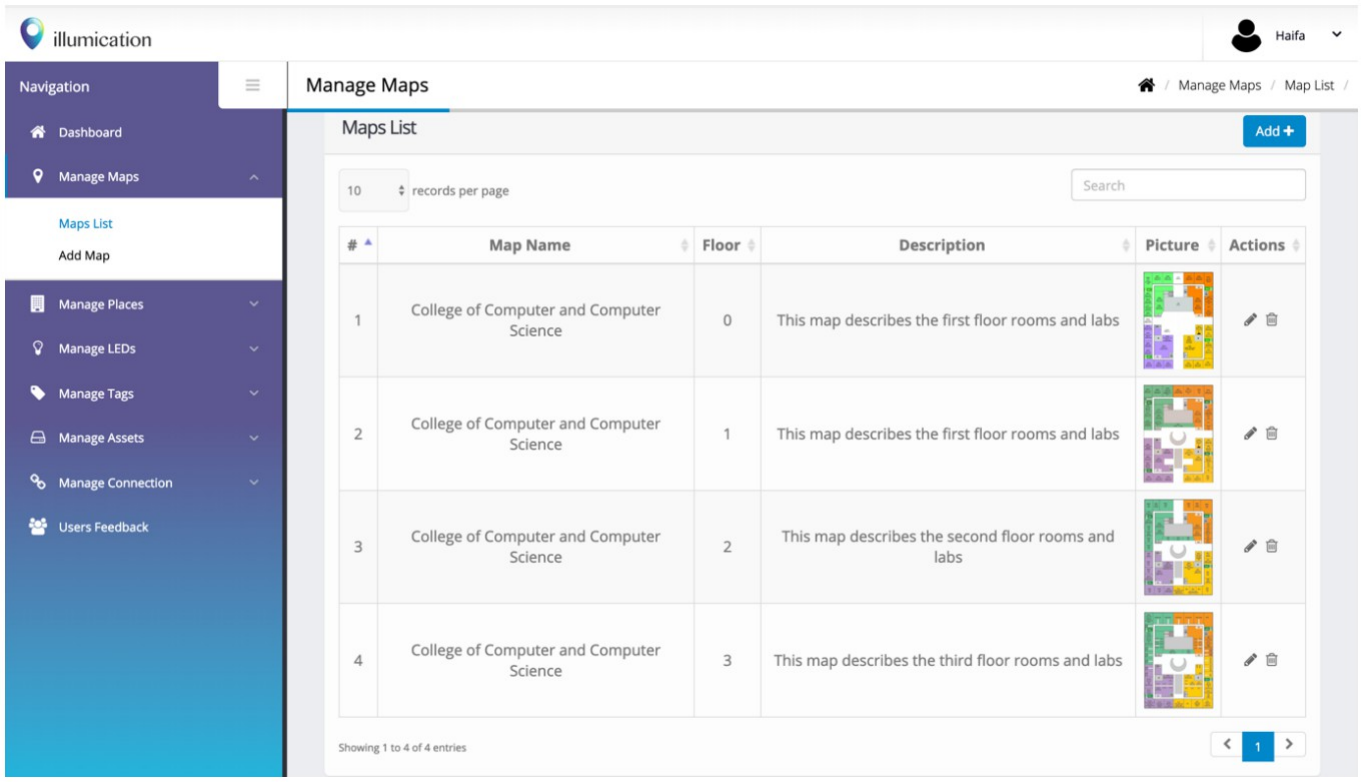
Web panel of the illumication system.

The proposed system, an asset localization system using Li-Fi technology (i.e., illumication), is used to test the proposed solution which consists of a mobile application and a web panel. In this phase, the administrator maps the actual locations of the LEDs to the system’s logical locations, connecting the tags related to the assets to the LED. We used the web panel for the system that includes different functions, as shown in Fig 5, to manage the maps, LEDs, BLE tags, and assets.

### 6.3. Testing phase

**Fig 6:**
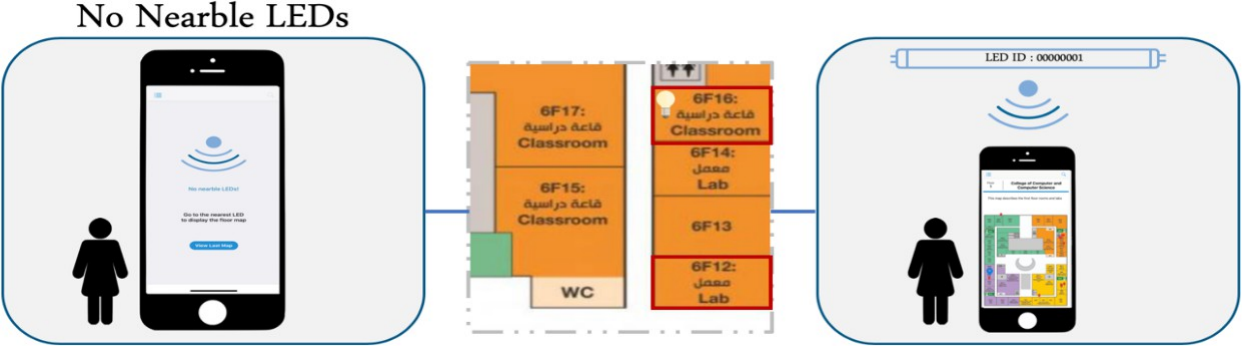
The user inside the light-emitting diode (LED) range.

**Fig 7:**
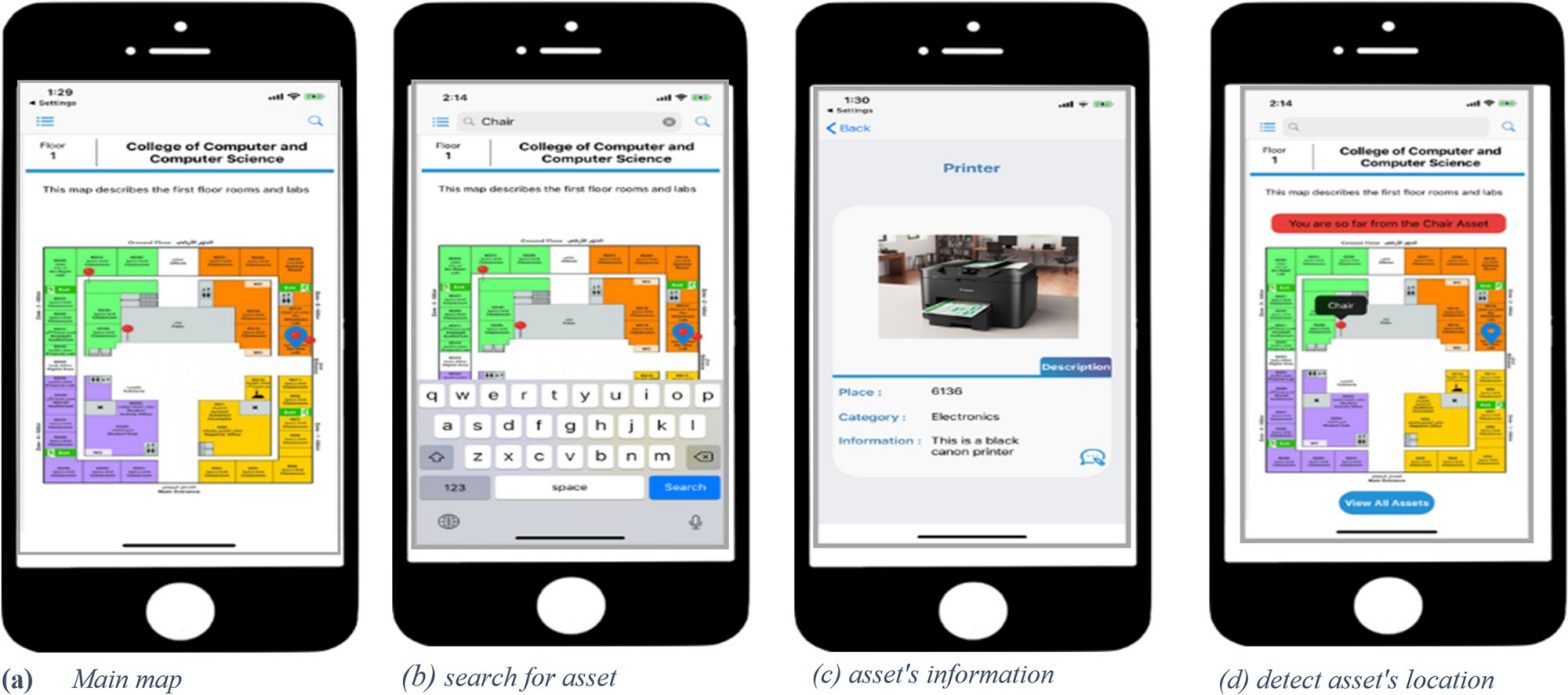
Mobile application of the illumication system.

Users are required to download the illumication mobile application for asset localization. As shown in Fig 6, when the user enters the building, the coverage range of the mobile phone detects the Li-Fi signal and shows the map. When the user starts the application, the map assigned to the nearest Li-Fi LED is shown, as in Fig 7a. Fig 7b shows the options available such as searching for the assets and viewing the assets. Fig 7c and 7d show that a user can see how far away the asset is based on their search.

It should be mentioned that the crowdsourcing function works in the background. It updates the asset’s location based on its tag and the LED light. The updating process depends on the RSS of the Bluetooth signal. Therefore, the asset’s location is updated only if the RSS is strong which means that the distance is very short, and therefore more accurate. This can decrease the signal inference and collision between the assets if they are all in the same place.

**Fig 8:**
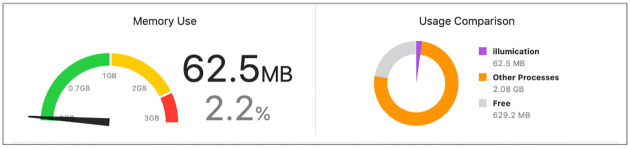
Performance testing.

For the performance testing, we used XCode powerful instruments tool that provides the developers analytical data about the system runtime behavior, to monitor the application performance. Fig 8 shows the testing of the overall memory use, by running the application for 15 minutes on iPhone X and comparing the results to other processes we found that it uses about 62.5MB which is 2.2% of the total iPhone memory 256GB. In addition, we have seen that the highest memory usage during the 15 minutes was 67.2MB and the lowest is Zero KB. Therefore, we can conclude that there is no unexpected growth or problems with the memory and it can be safely light on the devices.

## 7. Results and discussion

**Fig 9:**
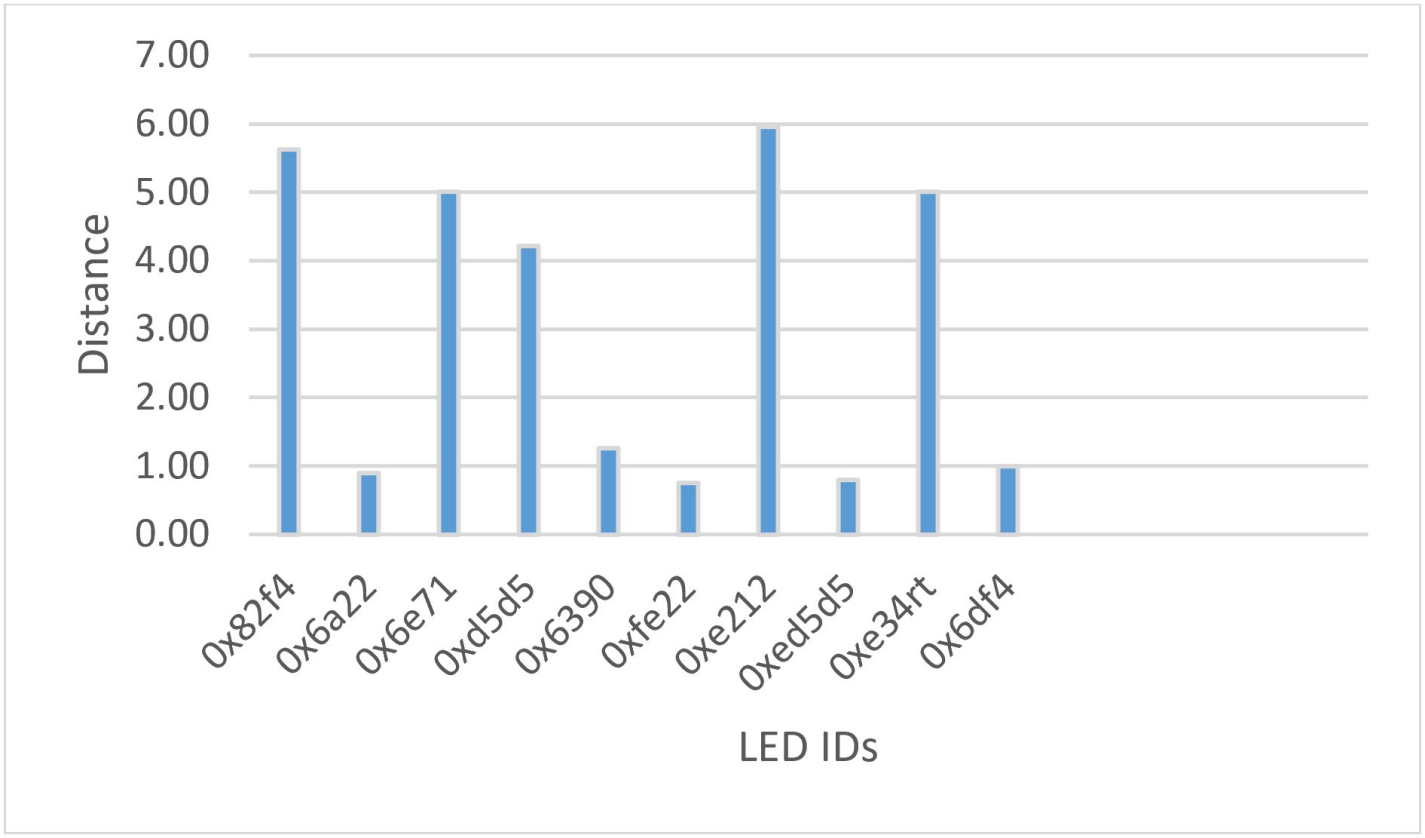
The LED IDs and the distance between mobile application and assets.

The experimental results show that the proposed system, which includes a web panel and location aware mobile application, can accurately locate an asset. It can detect the asset according to the LED locations in the building. Since the system links the BLE tags with the LEDs and updates the asset locations automatically if the asset locations change, the updating process which is done by crowdsourcing can work in close range of the assets. The mobile application receives the LED IDs, BLE tags and the distance from user to the asset. The zones around Estimote BLE stickers can be divided into far, almost near and near zones. The received signals strength can estimate the distance between mobile user and BLE tags that attached to asset. The system will show a suitable message to inform the user about the type of zone of the selected asset accordingly. It should be mentioned that the crowdsourcing function, which will be updating the asset’s location based on its tag will work according to the zones. To get more accurate result for updating the location, only the closer distance/zone should be considered. Therefore, the application will update the location according to the LED location only if the asset is really close. As shown in Table 3 and Fig 9, the system updates the assets to the new location (i.e., new LED ID) only if the distance is 1m or less and this number can be updated in the code if the updating process to only run at short distances.

**Table 3:** Crowdsourcing process.

Assets ID	BLE tags	LED ID	Location	distance (m)	crowdsourcing
C1	0b441d3579a2f443	0x82f4	Classroom1	5.62	no
C2	31931f37b10c4ca0	0x6a22	Classroom2	0.89	yes
C3	69eac5b8d9d6d039	0x6e71	Classroom3	5.01	no
C4	83470640c8fd1998	0xd5d5	Lab1	4.22	no
C5	885d18d88e162b42	0x6390	Lab2	1.26	no
C6	ad50123b84a7513d	0xfe22	Lab3	0.75	yes
C7	b10c4ca096e974f5	0xe212	Lab4	5.96	no
C8	c212413e31931f37	0xed5d5	Lab5	0.79	yes
C9	f2ds467e00f09ofi4	0xe34rt	Classroom4	5.01	no
C10	k823ud82e123f44e	0x6df4	Classroom5	1.00	yes

**Fig 10:**
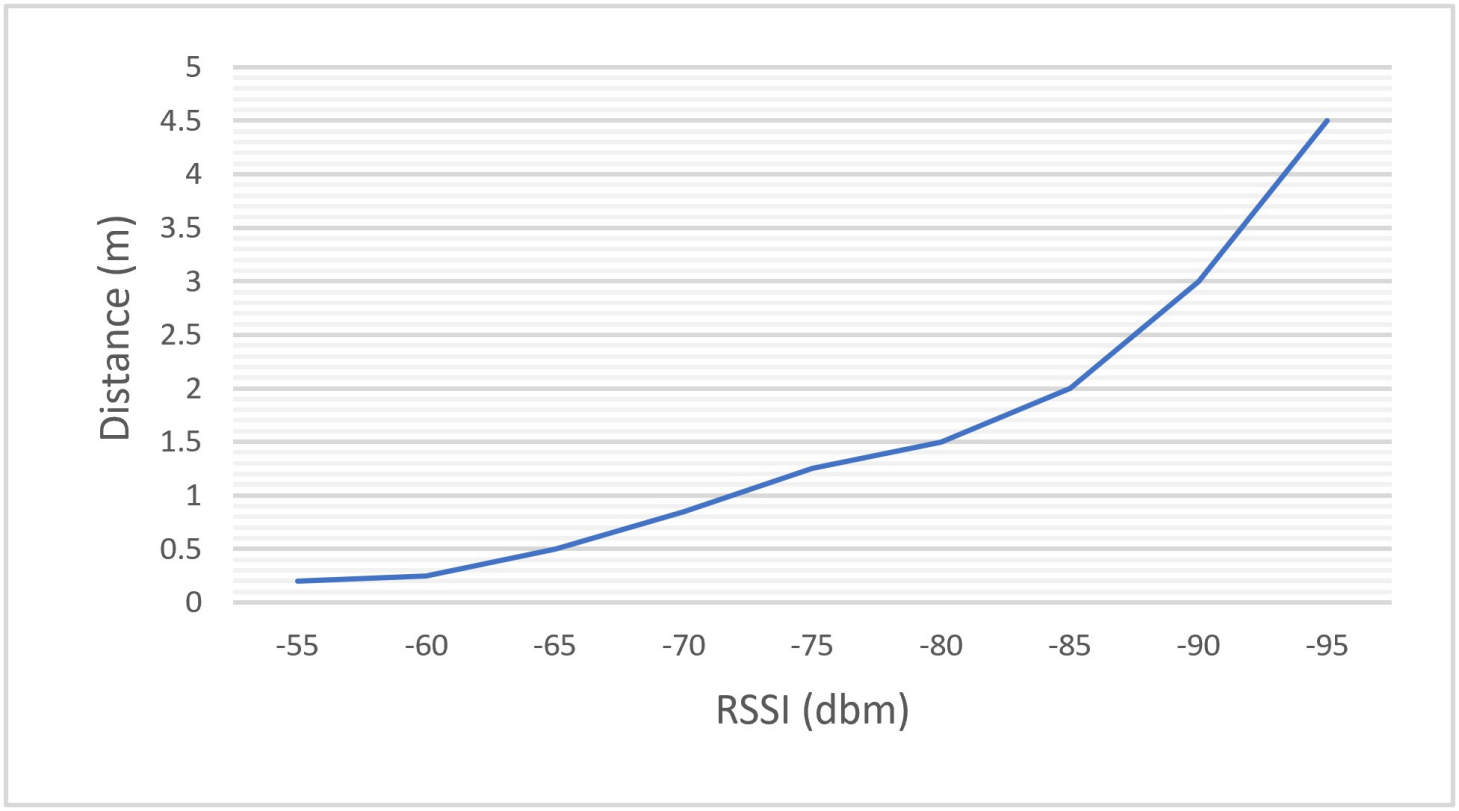
The received signal strength indication (RSSI) values of Bluetooth Low Energy (BLE) tags with their distances.

As shown in Fig 10, the estimated distance between BLE tags and mobile user depends on the received signal strength (RSS) which is actually reduced when the distance is increased.

**Fig 11:**
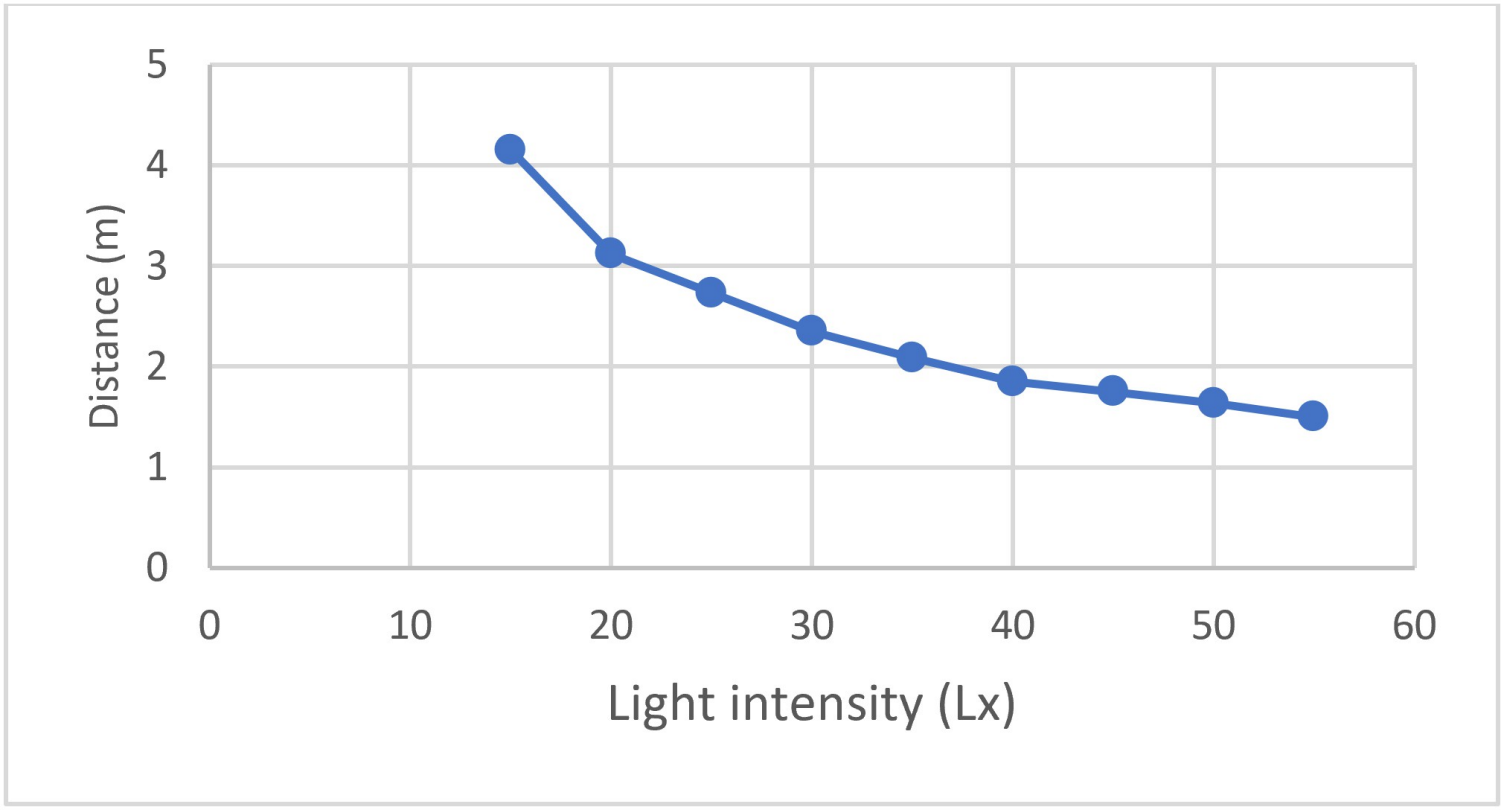
The light intensity (lx) and the distance between user and LED.

For LED lights, 14.8 W LED lights were used in this experiment and the distance to get the light signals depended mainly on the light intensity as shown in Fig 11. There are different measures in light, i.e., the lumen and the lux. On the one hand, the lumen is a measure of the total number of packets produced by a source of light. On the other hand, the lux is a measure of the intensity of light, that is, the number of lumens needed to illuminate an area, and it is equal to one lumen per square meter [33]. The lux can be calculated by using a Digital LUX Meter, which can measure up to 100,000 lux, or approximately 10,000 foot-candles. It is used for checking the level of luminance, i.e., the measure of the amount of light falling on a given surface [10]. Therefore, based on the result analysis, it is shown that whenever the lux is high it is more probable to detect the light and as soon as the lux becomes low the probability decreases until it does not detect the light. In the proposed experiment, the signal was detected at a value of 55 lx and greater which can be detected at 1.5 m and less.

**Fig 12:**
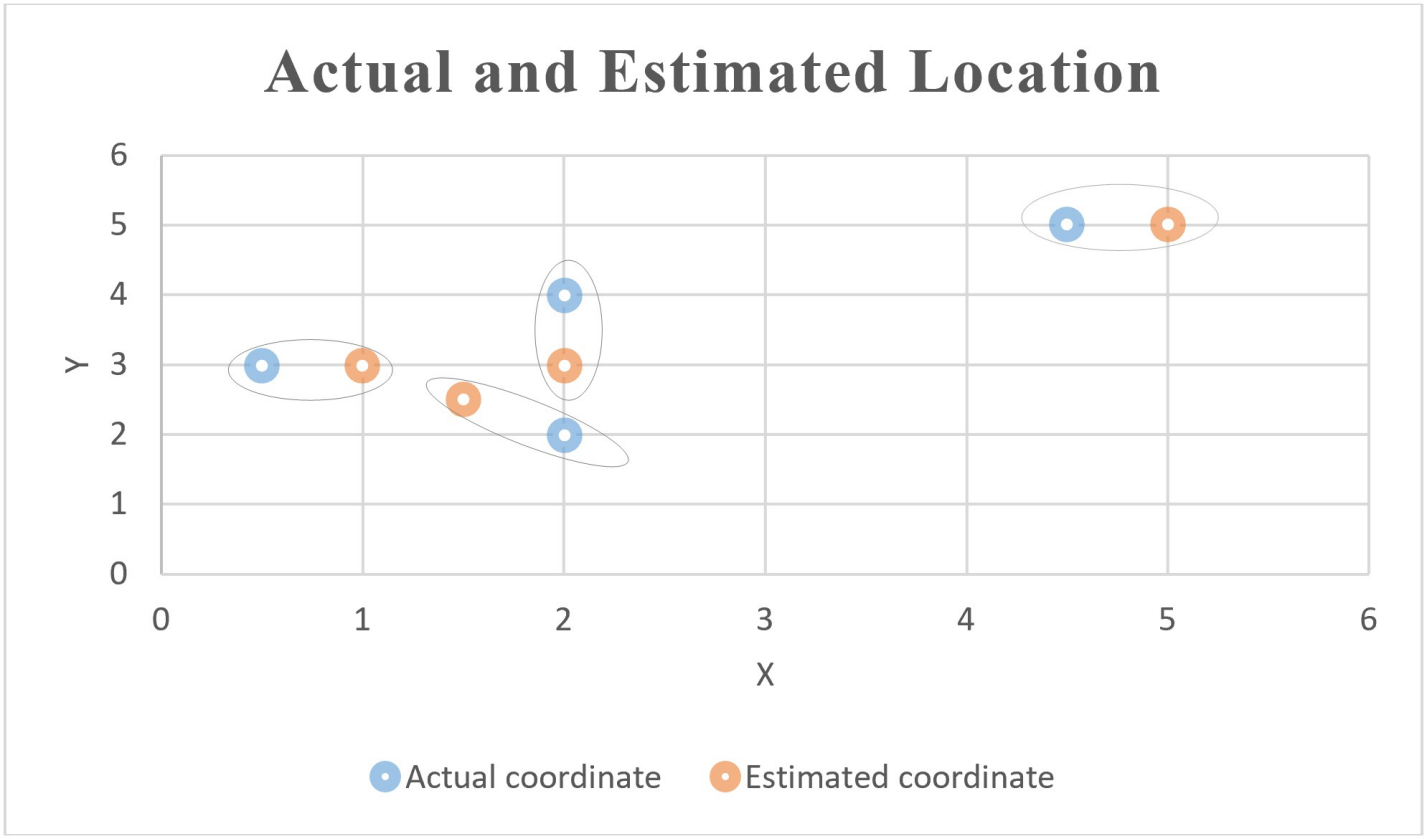
The different between the actual locations and estimated locations.

As previously mentioned, the system was tested for 10 LEDs and 10 assets, and a mobile application was used to identify their locations accurately on the map. For the updating process, four assets were changed to other rooms and updated to the correct locations. As shown in Table 4 and Fig 12, the different between the actual locations and estimated locations using the proposed approach are presented. The localization error is computed using the Mean Square Error (MSE). It should be mentioned here that there are different points should be noticed. The limited coverage range of LED, the light signals cannot pass the walls, and considering only the near Bluetooth signals that can be get in distance less than a threshold number all of these can improve the accuracy rate. In this study, we consider the distance that less than or equal 1 meter, but if we decrease this threshold, this will improve localization error significantly.

**Table 4:** The different between the actual locations and estimated locations.

Unknown Target Location	Actual location	Actual coordinate	Received RSSI	Distance from asset and mobile application (user)	Estimations by LiFi-based proximity technology	Localization error
C1	Classroom1	(1,1)	-99	5.62	distance > 1	-----
C2	Classroom2	(4.5,5)	-67	0.89	(5, 5)	0.50
C3	Classroom3	(2,5)	-97	5.01	distance > 1	-----
C4	Lab1	(3,3)	-94	4.22	distance > 1	-----
C5	Lab2	(2.5,3)	-73	1.26	distance > 1	-----
C6	Lab3	(2,2)	-64	0.75	(1.5,2.5)	0.71
C7	Lab4	(3,5)	-100	5.96	distance > 1	-----
C8	Lab5	(2,4)	-65	0.79	(2, 3)	1.00
C9	Classroom4	(4,1)	-97	5.01	distance > 1	-----
C10	Classroom5	(0.5, 3)	-69	1.00	(1,3)	0.50

## 8. Conclusions

Motivated by the importance of indoor localization and the recent interest in Li-Fi technology, the main goal of this study was to develop an asset localization system using light fidelity and BLE technology. The system utilized LED lights to detect the locations of available assets in a smart building, which allowed the administrator to manage the assets and LED lamps using a web panel and also enabled the user to search, identify, and share the location information of assets. In addition, the updating process of the asset localization was performed using crowdsourcing. We reviewed related works and similar applications, and provided a comparison between different applications that have been developed for indoor localization using Li-Fi technology in order to highlight the limitations that need more improvement. We also presented a real-world experiment using the proposed system and reported results of this experiment. It is shown that the utilization of LED lights that installed in any building can improve the asset localization since the assets locations will be linked with the locations of these lights. The coverage range of each light is limited and cannot pass the walls, so this can improve the accuracy. Also, the localization error of the proposed algorithm can be improved when the distance between assets and mobile users are decreased.

For future work, we recommend conducting more research on Li-Fi technology to support this research field. We also plan to provide a comprehensive analysis of the effectiveness of Li-Fi and BLE in indoor localization and compare them with other well-known technologies. In addition, we suggest testing the system and measuring the accuracy rate, and complexity analysis as well as confirming the best distance that should be considered for the crowdsourcing process. We are working now on another research that study the performance of combining Li-Fi and BLE using different algorithm.

## Supporting information

S1 File (XLSX)
